# Neural Network-Enabled Flexible Pressure and Temperature Sensor with Honeycomb-like Architecture for Voice Recognition

**DOI:** 10.3390/s22030759

**Published:** 2022-01-19

**Authors:** Yue Su, Kainan Ma, Xu Zhang, Ming Liu

**Affiliations:** Institute of Semiconductors, Chinese Academy of Sciences, Beijing 100083, China; yuesu@semi.ac.cn (Y.S.); makainan@semi.ac.cn (K.M.)

**Keywords:** flexible pressure and temperature sensor, artificial neural network, filament-microstructured, voice-recognition

## Abstract

Flexible pressure sensors have been studied as wearable voice-recognition devices to be utilized in human-machine interaction. However, the development of highly sensitive, skin-attachable, and comfortable sensing devices to achieve clear voice detection remains a considerable challenge. Herein, we present a wearable and flexible pressure and temperature sensor with a sensitive response to vibration, which can accurately recognize the human voice by combing with the artificial neural network. The device consists of a polyethylene terephthalate (PET) printed with a silver electrode, a filament-microstructured polydimethylsiloxane (PDMS) film embedded with single-walled carbon nanotubes and a polyimide (PI) film sputtered with a patterned Ti/Pt thermistor strip. The developed pressure sensor exhibited a pressure sensitivity of 0.398 kPa ${}^{-1}$ in the low-pressure regime, and the fabricated temperature sensor shows a desirable temperature coefficient of resistance of 0.13% ${}^{\circ }$ C in the range of 25 ${}^{\circ }$ C to 105 ${}^{\circ }$ C. Through training and testing the neural network model with the waveform data of the sensor obtained from human pronunciation, the vocal fold vibrations of different words can be successfully recognized, and the total recognition accuracy rate can reach 93.4%. Our results suggest that the fabricated sensor has substantial potential for application in the human-computer interface fields, such as voice control, vocal healthcare monitoring, and voice authentication.

## 1. Introduction

The human voice, as the main medium of communication with the outside world, plays a significant role in various aspects such as telecommunication, human-machine interaction, and the Internet of Things [1]. Conventional rigid microphones have been developed for accurately detecting human voice, but their applications are limited in noisy or windy environments. Recently, the flexible wearable pressure sensors that can provide electrical feedback in response to external pressure stimuli have been used for monitoring human voices [2,3,4,5,6,7,8,9,10,11,12]. By measuring vibrations in users’ neck skin and converting them into readable signals, these sensors have advantages of clear voice detection and anti-interference. In order to accurately obtain meaningful acoustic waveform, the sensors based on various transduction mechanisms have been proposed, including triboelectricity [2,3], capacitance [4], piezoelectricity [5,6], and piezoresistivity [7,8,9,10]. Among them, the sensors that rely on piezoresistivity have attracted much attention due to their simple device assembly and low energy consumption [11,12,13]. Although great achievements have been made in the fabrication of piezoresistive-type sensors, such as skin-conformity and technology compatibility, achieving high sensitivity in a wide measuring range remains a subject worthy of intense study.

In recent years, it was found that introducing various microstructure or nanostructure geometries, mainly including the molded bionic structure [14,15,16,17,18] and the regular ordered-array microstructures [7,19,20,21,22], into the piezoresistive-type sensors is a relatively effective method to achieve high sensitivity [11,18]. These microstructures can improve the compressibility of elastic materials, resulting in large contact resistance change between two conductive films when pressure is applied. However, the fabrication of these microstructures is based on the use of either complicated and time-consuming conventional photolithography techniques [7,19,20,21,22] or low-cost but low-customizable techniques that take advantage of certain naturally existing materials as molds [14,15,16,17,18]. There is, therefore, a need to develop a low-cost, efficient, and highly customizable techniques for sensor micro-structuration.

Several alternatives based on simple and low-cost techniques have been proposed, such as laser engraving [23,24] and pre-stretching method [11] so that the sensing performance of the devices could be improved in a quicker manner. Most recently, a robust approach to achieve high-reproductive micro-structured flexible films composing the sensors is developed by replicating honeycomb-like architecture from silicon molds that are produced by femtosecond laser pulses in the self-channeling regime [25,26]. Meanwhile, this fabrication approach is low-cost, ready for mass-production, and allows easy tailoring of the size of microstructuration, thus providing a meaningful strategy for introducing microstructures into the flexible sensor. Based on the specific structural design, high-performance flexible pressure sensors microstructured with femtosecond filamentation pulses are assembled and are used with success in detecting real-time artery waveforms and throat muscle movement during a speech [26]. Moreover, with the assistance of the principal component analysis (PCA) algorithm, the fabricated sensor can unambiguously distinguish different phonations, which preliminarily shows remarkable potential in voice recognition [26]. Although the fabricated sensor can unambiguously identify subtle vibration changes from the epidermis layer at the throat, voice recognition ability under a large amount of data still needs to be further verified.

Here, we demonstrate a flexible wearable pressure and temperature sensor to achieve accurate human voice detection, which is composed of the patterned Ti/Pt thermistor strip by magnetron sputtering, high-conductivity Ag thin-film electrodes, and honeycomb-like microarchitecture polydimethylsiloxane (PDMS) elastomers embedded with electrically conductive SWNTs (single-walled carbon nanotubes). The assembled sensor exhibits a sensitivity to pressure and vibration, which could achieve a pressure sensitivity of 0.398 kPa ${}^{-1}$ and a temperature coefficient of resistance (TCR) of 0.13% ${}^{\circ }$ C. A large number of resistance change waveforms were recorded by noninvasively monitoring vocal fold vibrations during the speech and input to a neural network model for further training and testing. An artificial neural network, as one of the most powerful machine learning methods based on data representation, has made a huge impact in a variety of application domains such as image segmentation [27], diagnosis [28], price forecast [29], and so on. By combining the neural network model with the fabricated device, we have successfully realized the recognition of vocal fold vibrations of different words during the pronunciation, and the total recognition accuracy rate can reach 93.4%. The above results mean that the developed neural network-enabled device exhibits remarkable application potential in the voice-recognition field.

## 2. Experiments

**Figure 1:**
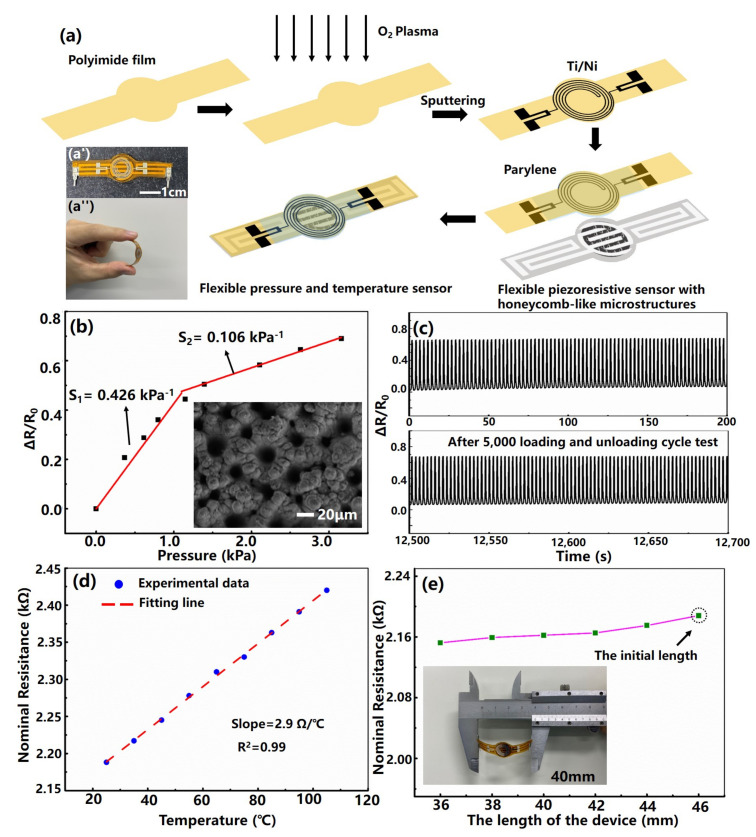
(**a**) Schematic of the preparation procedure for flexible pressure and temperature sensor with honeycomb-like architecture. Insets: (**a’**) Photograph of the flexible sensor taken by a digital camera. (**a’’**) The photograph of the fabricated device under bending. (**b**) Relative resistance changes under increasingly applied pressure. Inset: SEM image of the filament-processed silicon mold. (**c**) The stability cycling test of the pressure sensor under the pressure of 3 kPa. (**d**) The nominal resistance change versus the applied temperature. (**e**) The nominal resistance change of the temperature sensor under different bending states. Inset: Schematic diagram of the device length under a specific bending state.

**Sensor Fabrication.** Figure 1a illustrates a schematic of the fabrication process for flexible pressure and temperature sensor with honeycomb-like architecture.

*Fabrication of the temperature sensor:* A 200-micrometer-thick polyimide (PI) film was ultrasonically cleaned, successively, with acetone, ethanol, and deionized water and then dried with a nitrogen gun. Then, the PI film was treated with oxygen plasma to increase the surface adhesion. Next, a patterned mask based on the laser processing was placed onto the PI film, and then a 20-nm-thick titanium (Ti) film and a 100-nanometre-thick platinum (Pt) film were sequentially deposited on the surface of the PI film by sputtering. In order to protect the fabricated Ti/Pt thermistor strip, a 500-nanometer-thick parylene polymer film was vapor-deposited on the surface of the PI film. After that, the sample was placed on a hot plate to rapidly raise the temperature to 120 ${}^{\circ }$ C and naturally cooled to room temperature to improve the stability of metal-sensitive materials. Finally, the temperature sensor with a specific shape was obtained by cutting with an electronic film cutting machine.

*Fabrication of the pressure sensor:* Firstly, the honeycomb-like architecture silicon mold can be rapidly fabricated based on femtosecond laser filament processing technology, and then the PDMS films inked with micro-nano structures can be prepared. After that, SWNTs aqueous dispersion was drop coated onto the fabricated PDMS film surface and desiccated to fabricate a patterned SWNTs/PDMS conducting film, and the patterned Ag/PET thin film was prepared by using the printing process. Finally, the flexible sensing device was conducted with SWNTs/PDMS-conducting film and the Ag/PET thin film. The detailed fabrication steps of the device can refer to previous work [26].

*The assembly of the pressure and temperature sensor*: The edges of the obtained polyimide (PI) film sputtered with Ti/Pt thermistor strip and the flexible piezoresistive sensor with honeycomb-like architecture were bonded with 3M tape. The inset(a’) in Figure 1a exhibits the photograph of the fabricated flexible pressure and temperature sensor taken by a digital camera. In order to demonstrate the great flexibility of the sensor, the inset(a’’) in Figure 1a shows the photograph of the fabricated device under bending.

**Sensing Performance Measurements.** The performance of the temperature sensor was tested by a homemade test platform, which is composed of a source meter (Keithley 2612B system from a Tektronix company, Beaverton, Oregon United States) and a temperature controller. The test temperature is set from 25 ${}^{\circ }$ C to 105 ${}^{\circ }$ C, and 10 ${}^{\circ }$ C is used as a test node. After reaching the given temperature, keep it for 5 min, and use the source meter to test the sensor resistance value at this working temperature. To measure the sensors’ response to pressure variations, a force gauge was used to apply external pressures with a data acquisition instrument (Keithley DAQ6510) to detect resistance changes in real time. The bending test of the device was carried out on a motorized platform with a scale. Artificial neural network modeling uses Pytorch as the framework in the Python 3.6 environment.

## 3. Results

In order to characterize the pressure sensing performance of the fabricated flexible device, we investigated the relationship between resistance response and the applied pressure. Sensitivity (S), as a key parameter of the pressure sensor, can be defined as $S=(\Delta R/R_{0})/\Delta P$ , where ΔR denotes a change in resistance before and after a certain pressure is applied, $R_{0}$ denotes the pristine resistance value, and ΔP represents the change of the applied pressure. According to the test results in the previous work [26], the fabricated sensor exhibits a desirable compression sensitivity of 0.266 ${\mathrm{kPa}}^{-1}$ when the applied pressure is under 3.2 kPa, and a sensitivity of $4.02\times 10^{-4}$ ${\mathrm{kPa}}^{-1}$ over a wide range of pressures from 20 to 160 kPa. The presence of two different sensitivities in the consecutive pressure regions may be explained by the change in the contact area between the top and bottom patterned films. To further observe the sensitivity of the device in the low-pressure regime, we zoom into this range, as shown in Figure 1b, from which it can be observed that the sensing range can be roughly divided into two linear regimes with different sensitivity values, that is, 0.426 ${\mathrm{kPa}}^{-1}$ and 0.106 ${\mathrm{kPa}}^{-1}$ , for the pressure region of 0–1.2 kPa and 1.2–3.2 kPa. The existence of the two different sensitivities in the low-pressure range may be due to inconsistency of the size and depth of the honeycomb-like microarchitecture formed on the silicon surface fabricated by femtosecond laser filament, as shown in the inset of Figure 1b. In order to evaluate the durability of the fabricated pressure sensor, the device was applied with a stability cycling test under the pressure of 3 kPa at a 0.4-Hz repetition rate, and the result is shown in Figure 1c. It can be observed that the relative resistance change repeats with almost the same intensity and shape even after 5000 loadings and unloading cycle tests, revealing that the device exhibits high repeatability.

Furthermore, we measured nominal resistance change versus the temperature loading of the flexible temperature sensor, as shown in Figure 1d. Obtained data show that the device exhibits excellent linearity ( $R^{2}=0.99$ ), with a slope of 2.9 $\Omega {/}^{\circ }C$ . The temperature coefficient of resistance, as an important indicator of the temperature sensor, is defined as $TCR=(\Delta R/R_{0})/\Delta T$ , where ΔR denotes the resistance change before and after a certain temperature is applied, $R_{0}$ denotes the initial temperature value, and ΔT represents the change of the applied temperature. The fitted TCR of the temperature sensor is 0.13% °C for the range of 25 °C to 105 °C, which is comparable to that reported in the previous literature [30,31]. We further explore the mechanical properties of the flexible temperature sensor by measuring nominal resistance change of the device under different bending states, as shown in Figure 1e. It can be observed that the resistance value is approximately the same during the bending process, indicating that the device can be used on different occasions. The inset in Figure 1e exhibits the schematic diagram of the device length under a specific bending state.

## 4. Application on Word Recognition

**Figure 2:**
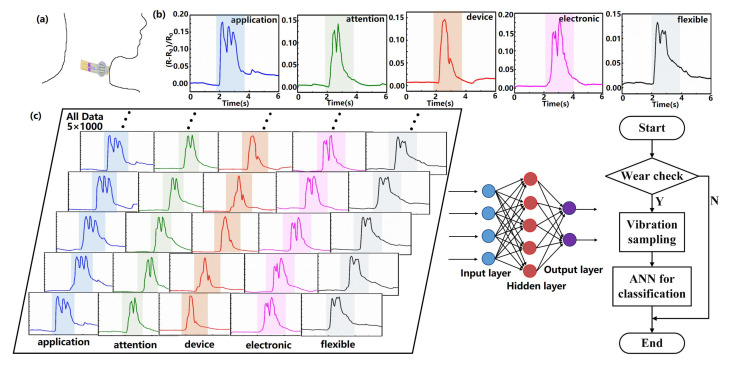
(**a**) Schematic diagram showing flexible pressure and temperature sensor attached to the neck skin for monitoring vocal fold vibrations during speech. (**b**) Resistance change waveforms of the fabricated device recorded for five words “application,” “attention,” “device,” “electronic,” and “flexible.” (**c**) The specific experiment flow chart.

Next, we attached the fabricated flexible pressure and temperature sensor to the tester’s (a 28-year-old Chinese male) neck skin for noninvasive monitoring vocal fold vibrations to explore the feasibility of the device in voice recognition (Figure 2a). Here, it should be pointed out that the influence of the tiny pressure caused by vocal cord vibration on the resistance value of the temperature sensor can be ignored. During the experiment, the measured temperature at the tester’s neck was 36.6 ${}^{\circ }$ C, which is in agreement with the result obtained by the electronic thermometer. The temperature (36–37 ${}^{\circ }$ C) is used to determine whether the device is worn for power management purposes, given that there may be some fluctuations in human body temperature. Incidentally, the temperature sensor needs a one-point calibration for different neck curvatures. As shown in Figure 2b, the obtained resistance change waveforms exhibited distinct patterns when a tester speak the words “application,” “attention,” “device,” “electronic,” and “flexible,” respectively. The above results show that the prepared high-performance device can distinguish different human pronunciations, and it is still unclear whether the recorded signal is sufficient for voice recognition. Therefore, we further explore the performance of the fabricated devices in speech recognition by combining them with the artificial neural network. In order to ensure that the neural network model can fully learn the features of each word and reduce the influence of human factors and the system noise of the sampling device, we repeatedly sampled the resistance change waveforms of five words “application,” “attention,” “device,” “electronic,” and “flexible” for 1000 times. Figure 2c shows the specific experiment flow chart. It can be found from Figure 2c that the waveforms of repeated tests with the same pronunciation show similar characteristic peaks and valleys, indicating the high reliability of the device.

Then, resistance data are sampled at 40 Hz. It should be noted that the sensor does not sample voice but the vibration of the vocal cord, which is caused by air from the lung passing through the vocal cord. Therefore, unlike the voice signal sampling requiring a high sampling frequency, the vibrations of the vocal cord are mainly on low frequency and can be sampled with a low sampling rate through the sensor attached to the neck. To balance the precision of sampling and the workload of data processing, the amount of the sampled data must be carefully considered. The higher sampling rate ensures a more precise signal, but it also brings a heavier workload of data processing. We have scanned the sampling rate from 10 to 100 Hz and found that 40 Hz is a proper sampling rate that can balance the precision and the accuracy of classification. The vibrations are transformed directly into changes of resistance with the pressure sensor and are measured 40 times per second, which is equivalent to a 40 Hz sampling rate. The advantage of this measurement is that it effectively avoids the disturbance of ambient sounds and the electrical noise of voice pickup equipment; thus, there is little noise.

**Figure 3:**
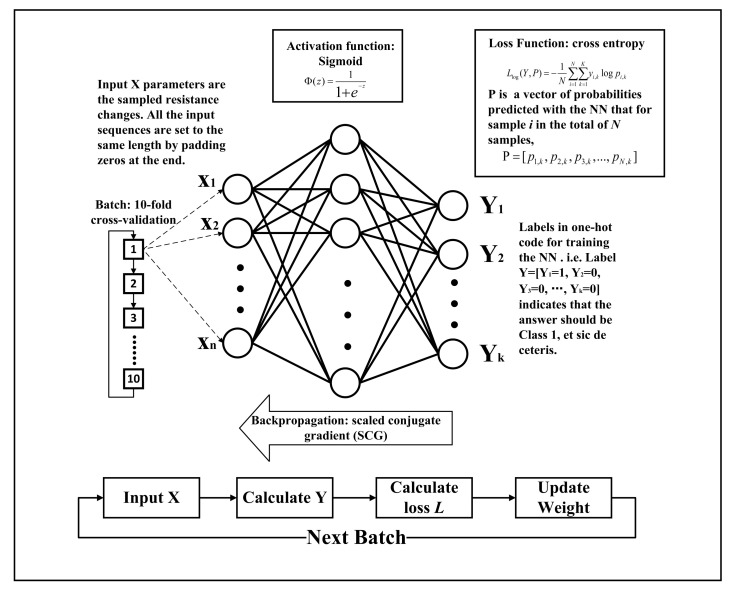
The training procedure of the proposed ANN. Training of the ANN is performed by using measured resistance changes data of the sensor.

The sampled data are directly used to train a typical three-layer fully connected perceptron for classification, which consists of an input layer (915 nodes), a hidden layer (five nodes), and an output layer (five nodes). In the dataset, the five words are labeled in one-hot code as class 1–5, corresponding to the output nodes of the perceptron. The input of the NN is the sampled resistance changes, and the targeted output is the one-hot encoded label of the vocabularies.The dataset is divided into a train set (70%), a validation set (15%), and a test set (15%). The train set is further divided into 10 batches to find the best hyperparameters. The idea of the 10-fold cross-validation is that 1 of the 10 is used as the validation set each time, and all the others are used as the train set. After 10 times of training, 10 different models are evaluated to select the best hyperparameters from them. The final model is obtained by using the optimal hyperparameters and all of the 10 as the train set to retrain the model. The loss function adopted is cross entropy, the activation function is sigmoid, and the backpropagation method is scaled conjugate gradient (SCG). The training of the supervised learning is shown in Figure 3. In each batch, the resistance change data **X** sampled from the sensor are input into the artificial intelligent network (ANN) to calculate a predicted output **P**. Then, **P** is compared with the target label **Y** to calculate a loss **L** with the loss function $L_{log}\left(Y,P\right)$ , which is then used to update the weights of the ANN with the SCG method. Instead of using random hyperparameters as initialization, after 10 batches of training, a set of the obtained hyperparameters that achieved the best performance is selected as the initial weights of the ANN for further training it with the entire train set. Namely, the aim of the 10-fold cross-validation is to find a set of hyperparameters having the best performance and to train the ANN based on the set to achieve a satisfying performance when the database is small. During prediction, the proposed ANN calculates the inferred output by multiplying input **X** with the matrix of the weights obtained during training.

**Figure 4:**
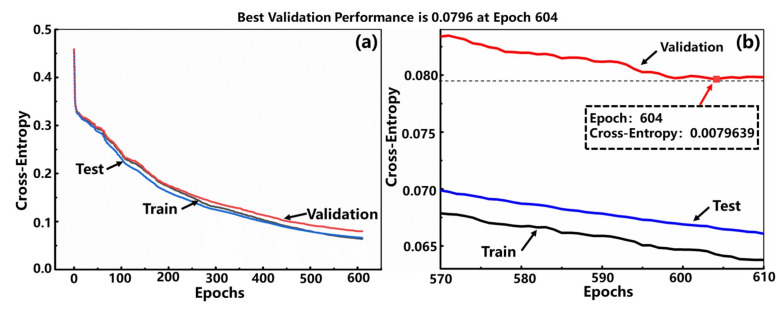
(**a**) The performances of the model training with epochs increasing. (**b**) The enlarged curves from epoch 570 to 610, where the best validation performance is at epoch 604.

**Figure 5:**
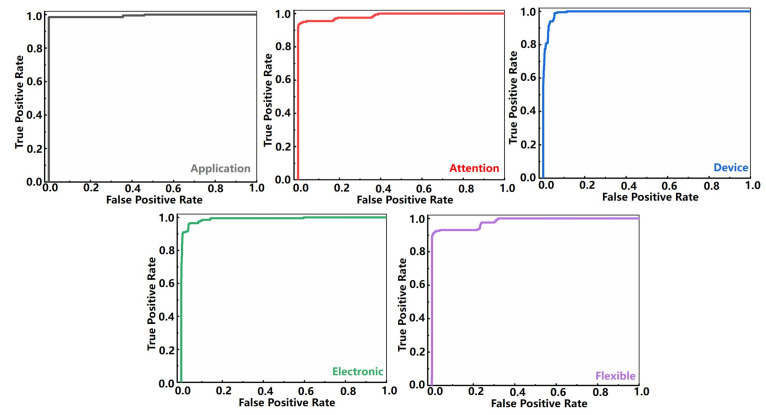
The receiver operating characteristic curve of the trained model.

Figure 4 shows the performance of the trained model. The downward trend of the curves of train, validation, and test is almost the same, meaning that there is no over-fitting problem. As the number of iterations increases, the loss gradually decreases, and the model converges. The best performance is achieved at the 604th epoch when the loss is the smallest (see Figure 4b). The receiver operation characteristic (ROC) curves in Figure 5 illustrates that the true positive rates (TPR) have already been greater than 0.9 when the false-positive rates (FPR) are less than 0.1. Namely, the correct classification rate (CCR) has already been very high even when the false positive rate is very low, which shows that the model is very sensitive to the input waveforms data of the words. Meanwhile, the area under the curve (AOC) of each word is very close to 1, indicating that the accuracy of the model is very high. Table 1 shows the confusion matrix of the classification results that the total classification accuracy rate of word recognition can reach 93.4% when checked with the validation set, which is relatively desirable compared with the previously reported literature with the stabilized value of 75% (an MXene-based sound detector combined with a more complicated deep learning network model for recognition the long vowels and short vowels of human pronunciation) [32].

## 5. Discussion

**Figure 6:**
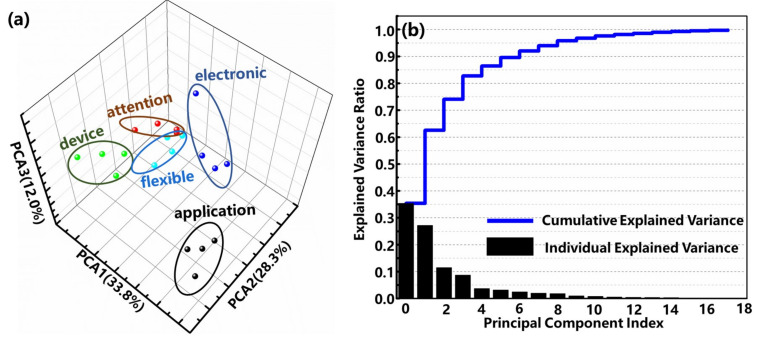
(**a**) PCA results of the randomly selected resistance change waveform data for the vocal cord vibrations in the coordinate system founded by the first three principal components. (**b**) Explained variance ratio of the randomly selected resistance change waveform data.

In order to explore the reason behind the obtained high recognition accuracy rate with a 3-layer perceptron, we conducted data analyses with the principal component analysis (PCA). With the PCA method, the acquired waveform dataset can be converted into a new space, which is composed of several principal components. The results of the randomly selected resistance change waveform data set by the PCA method are demonstrated in Figure 6. Figure 6a is drawn with the first three principal components, accounting for 74.1% of the total components (PCA-1 is 33.8%, PCA-2 is 28.3%, and PCA-3 is 12.0%). It can be seen that the sensor’s data from the same word are basically in the same cluster, and the clusters of different words are of a distance in space. That is to say, the principal components of the resistance change waveform of different words sampled with the sensor are of low correlation, indicating that the prepared high-performance pressure sensor is capable of accurately capturing the difference in the vibration of the vocal cords when different words are pronounced.

In word recognition tasks, the longer words contain more features, which usually require more complex network structures to recognize. The long words refer to those having at least two vowel syllables, relative to short words that contain only one vowel syllable, such as “cat,” “dog,” etc. Most word recognition tasks require complicated structures, such as convolutional neural networks (CNN) or recurrent neural networks (RNN), to realize. In this experiment, the vibrations are transformed directly into the changes of resistance with the pressure sensor, which effectively avoids the disturbance of ambient sounds and the electrical noise of voice pickup equipment; thus, the ANN can easily find patterns under little disturbance of noise. Therefore, a simple perceptron is sufficient to realize the recognition of long words with a good classification result with the help of the proposed sensor. Compared with voice signal processing, where filter banks, such as Mel-cepstral filter bank, are frequently used, voice filtering can be omitted, or at least the number of filters can be decreased with our sensor, which hugely reduces the computational workload of the on-chip system. Namely, pre-processing procedures of data cleaning, such as noise filtering, will be of little workload. Considering the fact that the sensor is designed for wearable devices, the reduction in computational workload can provide huge savings on power consumption. Therefore, the fabricated sensor and its simple-structured back-end neural network model is feasible for realizing the recognition of sound signals with lightweight preprocessing procedures, thereby exhibiting the substantial potential for application in a wearable device to assist voice recognition.

Regarding large-scale complicated word recognition tasks, PCA can be assigned on sampled waveform data for dimensionality reduction before inputting data into the ANN, which can efficiently decrease the size of the input layer, although it increases the computational complexity of the system. For example, as shown in Figure 6b, the first five components have already accounted for the dominant and the first 10 principal components account for 98.6% of all components. Therefore, the first ten components should be capable of representing a sampled waveform of a word, thus hugely reducing input dimensionality. Considering that the number of words is very large, the labels of vocabularies can be mapped to a fix-length hash sequence with a hash function to reduce the size of the output layer.

## 6. Conclusions

In conclusion, a wearable, flexible and neural network-enabled pressure and temperature sensor has been developed by integrating a PET film printed with the silver electrode, a filament-microstructured PDMS film embedded with single-walled carbon nanotubes, and a PI film sputtered with patterned Ti/Pt thermistor strip. The fabricated device could achieve a compression sensitivity of 0.398 kPa ${}^{-1}$ in the low-pressure regime, with a temperature coefficient of resistance of 0.13% ${}^{\circ }$ C from 25 ${}^{\circ }$ C to 105 ${}^{\circ }$ C, which has been demonstrated to be able to distinguish different human pronunciations and perform well in repeated tests. With the assistance of a lightweight artificial neural network, we have achieved the recognition of the vocal fold vibrations of different words with a total recognition accuracy rate of 93.4% without data pre-processing procedures. Analyses with the aid of the PCA method indicate that a good classification result is attributed to the capability of the fabricated device to accurately capture the vibration of the vocal cords. The above results suggest that with the continuous enrichment of waveform datasets, the fabricated sensor and its simple-structured back-end neural network model has significant potential as the next generation voice-recognition device for applications in human–computer interface and many other fields, such as voice control and vocal healthcare monitoring.

Our next step of work is to implement a wearable device based on the sensor for voice recognition and build a large dataset of vocal fold vibrations for the device.

## Figures and Tables

**Table 1:** The confusion matrix of the classification results (unit: %). Test Confusion Matrix, CCR = 93.4%.

Output Class	Target Class				
		1	2	3	4	5
**1**	18.9	0.6	0.0	0.0	0.0
**2**	1.3	18.7	0.6	0.4	0.0
**3**	0.2	1.9	15.3	0.8	0.2
**4**	0.0	0.0	0.4	19.9	0.0
**5**	0.0	0.0	0.0	0.4	20.7
